# Extracts and Gleanings

**Published:** 1876

**Authors:** 


1. Call the attention of the patients to the bad odor of their mouth, and inform them that not only in it, but also in the nose, there is something being secreted which poisons the whole system.

2. Place at their disposition an unlimited quantity of a solution containing two hundred grammes of decoction of barley, thirty grammes of honey, and twenty-five grammes of vinegar. Let them gargle and rinse their mouth with this frequently, and also snuff it into both nostrils. When they have commenced with this, it will be found so agreeable that large quantities will be consumed.

3. The nurses should be instructed to encourage and assist the patients during this operation. Where the adynemia is very profound, the nurses should cleanse the mouth for the patients. —*Gaz. des Hop.* and *Trib. Med.*, No. 308, 1874. G. R. C.

PROPHYLACTIC IN CHOLERA INFANTUM.—The numerous cases of gastro-intestinal catarrh occurring in small children during summer preponderate among such as are fed with the bottle. The various kinds of treatment adopted by physicians have not proved very successful, hence a phrophylactic against this disease is of great value.

As the affection originates in the nourishment of the infant, Jacusiel (*Berl. k. Wocherschrift*, 1875) has been led to add two tablespoonfuls of a one-third per cent. solution of salicylic acid in water to the daily allowance of milk, with the effect of rendering the germ of the disease powerless. The children fed in this manner have not had gastro-intestinal catarrh, or suffered any inconvenience from this rather free use of salicylic acid. The remedy is harmless and also inexpensive —*Hospitals-Tidende*, September, 1875.

COTTON WOOL IN THE EARS AS A PHROPHYLACTIC AND CURATIVE APPLICATION IN CORYZA AND SORE THROAT.—A correspondent to the *Practitioner*, February, 1875, says that, though specially subject to colds of unusually severe and oppressive character, he has for seven years been able to stave them off by the judicious use of cotton wool in his ears. Only the side of the nose or throat affected or exposed need be protected. The

Administration of Raw Meat.—Dr. Lailler proposes to mix 100 grs. of grated raw meat with 40 grs. of powdered sugar, added afterward 20 grs. of Bagnols wine (sparkling ?) and 3 grams of tincture of cinnamon. This mixture has an agreeable taste and is easily digested.—*Jour. de Phar. et de Chim.*, 1876, Nov., p. 367.

Solubility of Borax in Glycerin.—According to Gandolphe, glycerin dissolves, at the ordinary temperature, its own weight of borax by triturating them in a mortar, or more rapidly by applying the heat of a water-bath. This solution which keeps unaltered is well adapted for mouth washes after the addition of some clarified honey or honey of roses. 100 parts of water dissolve only 8.33 parts of borax. Boracic acid is likewise more soluble in glycerin than in water, but not to the same egree as borax.—*Ibid, from Union Phar.*

The Bromhydrate of Quinine.—Gubler.—(*Times and Gazette*, October 9, 1875.)—Terminating his account of the Therapeutical Employment of Bromhydrate of Quinine (*Journal de Therapeutique*, September 10,) Prof. Gubler arrives at the following conclusions: 1. The bromhydrate corresponding to the sulphate of the same base is more soluble and more rich in alkaloid than the latter. 2. It possesses the physiological properties of salts of quinine in general, and probably also the therapeutical virtues of its officinal congener. 3. Still the action of the bromhydrate seems to differ from that of the sulphate of quinine, not only by the moderation of the symptoms of quinic intoxication (*ivresse*,) but also by a marked tendency towards nervous sedation and hypnotism. 4. The qualities it thus possesses especially indicate its use in the treatment of the congestive and febrile affections of the nervous system—neuralgia neuritis, irritative neuroses, encephalic hyperæmia, etc.,—in combating which it has already furnished excellent results. 5. The bromide has manifested remarkable power in a case of incoercible vomiting, and has frequently been of service in cases ordinarily amendable to the sulphate, as in visceral or articular fluxions, whether diathesic or not, rheumatismal or gouty, and in symptomatic fevers *à frigore*, etc. 6. This new medicine has been given in quantities of from six to fifteen grains per diem, in doses of three grains, administered sometimes in the form of pill, and at others of hypodermic injection. 7. Injected into the cellular tissue, it has always proved absolutely inoffensive. In no case has the hypodermic injection of three grains of the bromhydrate (equivalent to about four and a half of the sulphate) been followed by the slightest inflammatory accident, neither redness nor tumefaction being visible next day around the seat of puncture. 8. This complete inocuity, joined to its greater solubility, constitute an incontestable superiority of this new combination of quinine, and recommend it wherever there is indication or necessity of administering quinine hypodermically.

SULPHATE OF CADMIUM IN ACUTE URETHRAL BLENORRHAGIA. Dr. Gazeau prefers this agent as an injection to sulphate of zinc, its astringent action being more energetic and persistent. In chronic blenorrhagia of some years' duration the sulphate of cadmium is useless.—*American Practitioner.*

ON THE USE OF HEAT IN METRORRHAGIA.—M. Noel Gueneau de Mussy.—(*Annales de Gynecologie*, July, 1875.—In many cases of metrorrhagia resisting the action of all classical means, the writer has used, according to the recommendation of Dr. Chapman, applications of water over the sacral region, as hot as the patient could bear them. The water ought to be renewed every two or three hours. In all cases, the hæmorrhage, even the most obstinate, ceased. Dr. Chapman used water at a temperature of 46° C. The writer used the water at a temperature even more elevated—*Chicago Medical Journal.*

VARIX.—Rigaud.—(*La France Medicale*, Sept.)—In operations for varicose veins of the spermatic cord and extremities, R. began by exposing the vein and cauterizing it with Vienna paste (Pruner, Strasburg, 1851.) He noticed a considerable shrinkage of the veins when exposed to the atmosphere, and was thus led to try exposure without cauterization.

The vein is simply laid bare and isolated from contiguous tissues by a bit of black ribbon, a strip of plaster, or an India-rubber sound. About the seventh day the vein is commonly found obliterated. It gives way, and the parts heal rapidly. One hundred and forty cases of simple varix of extremities, and nineteen of varicocele, were thus successfully treated. In one case, the internal saphenous vein was implicated, with severe phlegmon and other serious complications. Success was hardly hoped for, but the termination was quite as happy as in the other instances.—*Chicago Medical Journal.*

THE TREATMENT OF DIFFERENT FORMS OF HEADACHE.—W. H. Day, M.D.—(*Lancet*, September, 1875.)—This is an article called forth by a previous one on the same subject by Dr. Moxon. Dr. D. objects to the classification of sick headache, as depending on the digestive system. "The nervous system is primarially at fault," and the digestive disturbance is a consequence. In persons predisposed to it, overwork, fatigue, late hours and excitement bring it on when no derangement of the digestive organs exists. Some young women with dysmenorrhœa suffer at every menstrual period. The author favors the use of bromide of ammonium and iron tonics, taken for a long time, to modify the nervous sensibility.

The headache occurring in many children who are debilitated, who have some hereditary cachexia, in those who show an aptitude for learning, and who, after any mental effort, become listless and variable in temper and manner, sometimes dull, at others fidgety and quarrelsome, with weak pulse and frequent sighing and yawning, and poor appetite—this headache he thinks is due to some "intricate change in the cerebral membranes or tissues of the brain." The headache may last for months together, and is aggravated by rigid discipline, moral or physical.

Bromide of potassium is the remedy, and must be continued for a long time, and be followed by cod liver oil and other tonics; iodide of potassium is often a valuable addition. He agrees with Dr. Moxon, that when, with headache, sickness of stomach and prostration are extreme, the extremities are cold and pulse feeble, and there is intolerance of light and sound, and the patient has been for days without relief, it is proper to inject, hypodermically, one-sixth of a grain of acetate of morphia.

This is often followed by perfect relief. The addition of the 120th or the 80th of a grain of atropia may counteract the tendency to nausea which morphia alone sometimes excites—*Chicago Medical Journal.*

CROTON-CHLORAL is another nerve sedative recently introduced, to fill the indications for which the hydrate of chloral and the bromides are so frequently given, and where they have failed. The *Amer. Jour. of Med. Sciences*, April, 1874, quotes Liebreich's experiments of the preceding year. The ingestion of drachm j. produced a deep sleep in fifteen minutes, with anæsthesia of the fifth pair of nerves. The tonicity of the facial muscles was unaltered. When it is administered as an hypnotic in mania, the patients fall asleep in a sitting posture, while the pulse and respiration remain unchanged. If an equal degree of anæsthesia had been produced by hydrate of chloral, the patients would have fallen from their chairs, and both pulse and respiration would have been retarded. Liebreich considers croton chloral indicated, therefore, when very large doses of the hydrate are required to produce sleep, or when it is altogether inapplicable on account of heart disease, or finally, as a successful substitute in the treatment of trifacial neuralgia. In tic douloureaux is only a palliative.

Bunsun Baker reports, in the same number of the *British Medical Journal*, five cases of neuralgia cured by one grain doses of croton-chloral repeated every hour during from three to six hours. Two of these were facial neuralgias, one from concussion of the spine, one neuralgia and dysmenorrhœa, one general neuralgia.

Sidney Ringer recommends the croton-chloral (*British Medical Journal*, Nov., 21, 1874) in five grain doses for the relief of sick or nervous headaches. A stupid feeling that is often left afterwards is easily relieved by bromide of potassium. — *Virginia Medical Monthly.*

ACIDULATED GARGLES IN TYPHOID FEVER.—M. A. Netter, alluding to the buccal element in typhoid fever, and the beneficial influence of frequently-repeated acidulated gargles, draws the following conclusions :

beneficial effects are most marked in persons with a large meatus, or a thin, delicate ear. Sore throats and colds on the chest are also greatly benefitted by its use, If it be remembered that irritation deep in the meatus will produce irritation and congestion of the throat, it will be easy to see the relation between the throat and external meatus, and how cold air in the external meatus may produce a sore throat. Protect your ears from continued cold winds or drafts, is the plain teaching of the above facts.—*Detroit Review of Medicine.*

Cantharidal Strangury.—Starr.—(*Practitioner.*)—Dr. L. E. Starr says that cantharidal blisters, moistened before application, with alcohol, will produce no strangury from the absorption of the active principle of the fly. Repeated trials with the above named result uniformly, led him to give this fact to the profession.—*Chicago Medical Journal.*

Use of Elecitricity for Hyperpyrexia in Typhoid Fever.—Gluk.—(*Annal: Univ. de Med.*, 1875)—In thirty cases, where the temperature exceeded 102 F., the author used the following means: He applied the positive pole from a pile with constant current upon the third cervical vertebra, and the negative pole upon a level with the superior cervical ganglion of the great sympathetic. Without going into details, he says there was *always* a considerable lessening of the temperature.

Syrup of Tolu.—Regarding the resin tolu as the main or soul active principle, Henrotte is in favor of retaining it in the syrup, snd effects its permanent emulsion in the following manner: 10 grams of finely-powdered tragacanth are triturated with sufficient simple syrup to form a mucilage; 40 grams of tincture of tolu are added and an emulsion made, to which enough simple syrup is added to make the whole weight 1,000 grams.—*Jour. de Phar. d'Anvers,* 1875, p. 337, 339.

Neuralgia.—This disease is essentially one of nervous debility. Any habit or pursuit which has a tendency to exhaust the nervous force, renders the individual peculiarly susceptible to neuralgic attacks.

In this disease phosphide of zinc exerts marked effect, producing a restorative influence upon the nervous centres and branches seldom witnessed by other remedies, and its curative effects upon exhausted nerve tissues is worthy of all considera-tion. One-tenth to one-half a grain repeated every two to three hours in pill form, made up with flower and simple syrup and sugar-coated, is the most eligible manner of administration, the stomach tolerates the medicine well, and I have never witnessed any unpleasant results from its use.—*Journal of Materia Medica.*

THE following pill is said to be invaluable in Chorea:

| | | | |
|---|---|---|---|
| R | Phosphide of zinc | gr. | x. |
| | Alc. ext. cimicifuga | gr. | xxv. |
| | " " hyoscyami | gr. | xx. |

M. ft. pil. no. L.

Sig. One to two pills three times a day, for a child twelve years of age.

SPINAL IRRITATION.—As the faculties of the spinal cord are built up by organization, so must they be kept up by due nutri-tion. If not so preserved in vigor—if exhausted by any means—ill effects are manifest in degenerate action; therefore spinal ir-ritation is in fact a mal-nutrition.

The differential diagnosis between spinal irritation and spinal congestion or inflammation is one, purely of degree and effect.

The following pill has proved curative in a number of very stubborn cases:

| | | | |
|---|---|---|---|
| R | Phosphide of zinc | gr. | x. |
| | Phosphate of iron | gr. | lxxx. |
| | Quinine | gr. | xl. |
| | Alc. ext. hyoscyami | gr. | xxx. |
| | Alc. ext. belladonna | gr. | xii. |

M. ft. pil. no. L., and sugar coat them.

Sig. One pill every four hours.—*Journal of Materia Medica.*

TREATMENT OF CHOREA BY ARSENIC IN LARGE DOSES.—Dr. Eustace Smith, in a note to the *British Medical Journal*, of May 1st, 1875, emphasizes the value of arsenic in chorea, but states that it was not so generally known that the curative value of the drug is greatly increased by administering it in full doses. Children ha[illegible] a remarkable tolerance for it, especially in such a non-febrile affection where there is no increased irritability of the digestive organs. To a child between the ages of five and six and twelve, he would give, in this complaint, as much as ten minims of Fowler's solution three times a day, directly after meals. The influence of the treatment is seen almost immediately, and it is rare for any of the physiological effects of the drug to be seen. Under this treatment, he says that severe cases seldom last longer than a fortnight.—*Jonrnal of Materia Medica.*

Hair Tonic.—Dr. J. N. Nowlin, of Georgia, sends us the following prescription, which he has used for years, and "has yet to meet the first instance of failure to arrest the falling off of the hair." He requests those who use it to report through this journal:

R Borax, powdered....................two drachms
Cologne water.........................eight ounces
Bay rum................................six ounces
Tinct. cantharides,
Spir. ammon. aromat., aa one ounce. M.

Sig. Apply to the scalp every morning, by thoroughly rubbing in.—*Philadelphia Medical Reporter.*

Enlarged Prostate Mistaken for Calculus.—M. Ripault, of Dijon, was called to assist at an operation for stone. The patient was adult, in whom all the usual symptoms of calculus were present: the characteristic sound occasioned by striking the sound against a hard body was distinctly heard by several persons, and the existence of a stone undoubted. The lateral operation was performed; the bladder was opened, but the forceps, several times introduced, brought away nothing but clots of blood. An encysted stone was suspected, but the operation was discontinued. Nevertheless, the pains which the patient previously experienced, ceased for some months, after which they recurred with greater violence; difficulty of micturition increased; the urine became ammoniacal; fever set in; and the patient died six months after the operation. On post mortem examination the prostate was found enlarged, composed of tough fibrous tissue, very horny on section, and when struck with a sound, gave the same sensation as was experienced during life. —*Medical Times and Gazette.*

Subcutaneous Injections of Stryconia for nervous Deafness.—Dr. R. Hagen, in the preliminary report of his experiments on these cases, published in the *Centralblatt fur die Medicinischen, Wissenschaften,* Aug. 14, 1875, says: "I have heretofore published an account of my success in the treatment of some cases of amaurosis and amblyopia by means of injections of strychnia. Since then, I have employed the same treatment in a series of corresponding cases, and after more than a half year's continued observation, I can say, without fear of contradiction, that the results are better and of longer continuance than those following any other mode of treatment. I have usually employed a one per cent. watery solution of nitrate of strychnia and made two injections weekly under the skin, in the vicinity of the mastoid process. In the majority of these cases, I made use of no other mode of treatment.

The discussion of the general as well as the special effects of these injections on the individual systems I reserve for a more extended article, which I hope soon to publish. These injections seemed to exert no effect whatever over subjective noises.

Carbolic Acid and Hyposulphites in Scarlatina.—Dr. Bland (Trans. Med. Soc. State of Pennsylvania) states that his results in the treatment of scarlatina with these remedies is unprecedented. He applies the carbolic acid with glycerine topically to the fauces, etc., and gives hyposulphite of soda internally, together with nutritious diet and febrifuge mixtures as required.

Digitalis in Bronchitis.—D. J. Braithwaite (*British Medical Journal*) strongly recommends the use of digitalis in the treatment of bronchitis accompanied by debility. This form of bronchitis is most frequent in persons past the meridian of life. If neglected, the patient soon presents the following symptoms: perspiring skin, poor, quick pulse, urgent dyspnœa, bluish tinge of lip and skin, and respiration accompanied by loud wheezing sounds.

A Hint in Giving Iodide of Potassium.—A useful hint is revived in the *British Medical Journal* by Mr. Joseph P. McSweeny. He says: "Sir James Paget was the first to call the attention of the medical profession to the following interesting fact, namely, that carbonate of ammonia greatly increases the therapeutic action of iodide of potassium. I have had extensive experience in the treatment of syphilis, and have tried it with the best results, and find that five grains of iodide of potassium, combined with three grains of carbonate of ammonia, are equal to eight grains of the potassium salt administered in the ordinary way."—*Chemical Gazette.*

Methods of Operating in Anal Fistula.—M. Jules Felix, of Brussels, employs a ligature of stout English silk, one end of which is passed through the anal opening. The ligature is then moved backward and forward with a see-saw motion, cutting its way rapidly through the intervening tissues until a complete section is made, as with the ecraseur, without the loss of a drop of blood.

Syrup of coffee is excellent for disguising the taste of iodide of potassium, and makes the use of this valuable remedy agreeable to the sick. The syrup of lemon is likewise very effectual. —*Clinical Record.*

Nitrite of Amyl in Neuralgic Affections.—*The Practitioner* contains letters relative to the curative effects of inhallations of this drug, from Dr. George H. Evans and Dr. R. A. Douglas-Lithgow. The former reports three cases of facial neuralgia in anæmic females in which marked, and in one case permanent, relief followed the use of the remedy. "In one instance a sister of one of the patients, incautiously, who was not anæmic, one day, sniffed at the amyl bottle, probably because I had especially cautioned my patient against allowing any of her sisters using it; and immediately her face became most painfully flushed, and she felt sufficiently uncomfortable to prevent her repeating the experiment. I think from what I have seen of its use, that anæmic people, as one might expect, can bear very much larger doses of amyl than those who are not anæmic."

Dr. Douglas-Lithgow has employed nitrite of amyl since 1869 for the relief of nervous cephalalgia, causing the patient to inhale a couple of drops from the palm of the hand. In several instances the dose has had to be increased or repeated. When due care had been exercised in the selection of cases, he had never found the remedy fail to produce entire and almost immediate relief.

Eserine Discs in Tic.—Dr. Munro, in a note to *The Lancet* of September 25th, describes a new and agreeable excipient for administration of eserine (physostigmine), the active principle of Calabar bean. He finds that one twenty-fifth of a kilogramme (.0006 grain), when put up in gelatine squares or discs, answers the purpose, one-half removing the tic, if it has no persistent cause, in from five to fifteen minutes. The discs are made by Duquesnel, of Paris. Dr. M. also finds that the bean, in the form of the extract, is of advantage in incipient bronchitis, congestion of the liver, and phthisis, with hot, dry skin; in sufficient doses, which, for an adult, are at least from one-fifth to one-sixth grain, he finds the temperature reduced from two to three degrees, the dose being repeated every four or six hours, under careful watching, until the desired effect is produced.—*Medical Record.*

Novel Treatment of Diabetes Insipidus.—Dr. Kerr (*Canada Lancet*) reports three cases of this affection which he treated successfully, by recommending his patients to introduce the catheter each time, immediately after they had micturated; for several days they succeeded in drawing off as much urine as they had passed voluntarily. When this was done at night, they were scarcely ever compelled to rise before morning. The quantity of urine speedily fell to the natural standard. Dr. Kerr suggests that possibly, in diabetes insipidus, the bladder never empties itself, and that the irritation produced by the residuary urine may, perhaps, share in causing increased secretion.

Chloroform in Malaria.—Dr. Joseph Bernes reports, in *The Medical Times*, four cases in which good results followed the internal administration of chloroform in "chills and fever."

He calls attention to the absolute desuetude into which this remedy has fallen, together with the incontrovertible fact that the potency of quinia is not invariably sustained.

In the cases reported the remedy was given in twenty drop to drachm doses hourly for three to six hours preceding the expected attack.

TREATMENT OF DIPHTHERIA.—In the mild cases, all the treatment I have resorted to, is to unload the bowels and give a standard solution of chlorate of potassium as a gargle, and tonic doses of quinia. But if the cases go on to the formation of a false membrane, I invariably place the patient under tinct. ferri mur. and quinia sulph. Apply tinct. ferri. mur. to the membrane, with the sponge probang, and continue the potas. chlor. gargle. I keep up the inhalation of steam from one quarter to half an hour, and, in very grave cases, for hours without intermission. I have discontinued the use of the ordidary inhaler, and form an extempore one for each patient. I take a double sheet of foolscap paper, and having made it in the form of a cone, fasten the apex around the spout of an ordinary tea-kettle, and, when steam is being well generated, have the patient's mouth brought to the edge of it, bringing it closer and closer, until the base of the cone surrounds the mouth and face. The patient, while getting a sufficiency of steam, likewise gets plenty of air. I have seen little ones breathing laboriously, quiet down in fifteen minutes after commencing inhalation. In those cases that do not vomit freely, I give syr. ipecac. with the hope that if the membrane is not dislodged, the throat, stomach, etc., may be applied to the putrid discharge, continually taking place from the diseased part. Formerly, in the commencement of the epidemic, I used sulphate of iron, but found by using the sulphate in one case and the tincture in another, that the membrane was loosened and separated more quickly with the latter drug. A number of times when the little one appeared sinking rapidly, I have removed the membrane with the sinking forceps, oft-times to the immediate relief of the sufferer, and if hemorrhage resulted in any large quantity, was soon able to stop it by the application of iron. From the commencement to the close I supported the strength as much as possible by beef tea, broths, and whisky toddy. It is wonderful what large quantities of stimulant these cases will bear, and how well many take it, even to dying. The external local application in mild cases, is simply the application of some household rubefacient, while in the grave cases I resort to sinapisms to the throat, reaching from ear to ear, followed by hot fomentations of hops, or hops and bread. In all serious cases, I keep up the warm baths from twelve to fifteen minutes, repeated several times during the day, while pediluvia and draughts to the feet and hands are used. To sum up: iron and quinia, with plenty of whisky, beef, tea, broths, with local applications of iron and gargles of potas chlor., inhalation of steam, hot baths, hot poultices to the neck, and hot pediluvia. Some may think this is doing too much, but I have oft times omitted one or more of them, and invariably found my patient not so well. Were I called upon to select a more simple treatment I would say hot baths, inhalation of steam, and plenty of whisky.—*Canada Lancet.*

Treatment of Typhoid Fever by the Cold Bath.—The recently published Transactions of the Medical Society of the Paris Hospitals, contains a suggestive and practical paper on this subject by Dr. H. Libermann. The author, who is surgeon-in-chief of the military hospital of Gros-Caillou, acknowledges that his attention was first drawn to the value of this method of treating typhoid fever by the success with which he had observed it to be pursued by the German ambulances during the war of 1870. He then gives a full historical sketch of the bath treatment of fever, assigning to Currie the honor of its introduction, and giving full credit to the good work done by the German physicians, notably Brand, Ziemssen, and Liebermeister. His own experience has at present extended over twenty cases, two of which were fatal, but he cannot adopt in its entirety the confidence that Brand has in the use of this valuable therapeutic aid. He gives, however, several statistics showing that the death-rate has been considerably reduced by the use of the bath (to nearly one-third of its previous scale), and this from a large number of cases, with death from all causes. The most marked reduction, however, has been in those cases which are fatal from the pyrexia itself The plan of treatment pursued by Brand is now well-known in this country. It consists in the systematic employment of the cold bath (63 degrees to 70 degrees Fahr.) for a time lasting from fifteen to twenty minutes three or four times daily, during which the temperature of the body is reduced from 2 degrees to 7 degrees, the amount of reduction being generally in proportion to the slightness of the case. The warm bath is indicated in cases of extreme nervous prostration, with feebleness of heart's action, or where there is much diarrhœa. The effects of the warm bath are, of course, not so striking as those of the cold, but both methods are marked by their sedative action on the nervous system, and the disappearance of dichrotism from the pulse. Dr. Libermann then enters fully into the indications and contra-indications for the use of the bath, which he says should be reserved for severe cases—a reservation incompatible with the early adoption (in the first week) of the treatment by Brand, a plan which has doubtless influenced that physician's statistical results. He believes the bath should be employed if, at the end of the first week or the beginning of the second, there is a high evening temperature with but slight morning remission, assigning a temperature of 104 degrees (in rectum) as the usual limit; or, if on any one occasion it should reach 105.8 degrees; or, if the remissions for days together do not exceed one degree. The bath is contra-indicated in slight cases, or in cases complicated with severe diarrhœa or symptoms of perforation of bowel, or where the rise of temperature is obviously due to collateral causes. Pneumonia and hypostatic congestion are benefitted by the bath, owing to the improvement effected in the vigor of the heart's action. He appends a few cases, and the whole essay, which is well worthy of perusal, is a gratifying indication that some Frenchmen, at least in medicine, are not slow to learn what is valuable from those who politically are their opponents —*Lancet.*

Case of Rheumatic Hyperpyrexia Cured by One Cold Bath.—Dr. Sidner Ringer reports the case of a young woman, aged eighteen, who had been the subject of an attack of acute rheumatism. On the fifth day of her illness, the temperature was 102.8°, and remained between 102° and 104° for four days, when the fever gradually declined. She had pericarditis and endocarditis, and an attack of pleurisy with slight effusion on the left side. On the eighteenth day the fever had almost gone, and although she had not left her bed, she was considered quite convalescent. On the nineteenth day, however, the temperature rose to 103.6°; and the same night it was 104° Early on the twentieth day, it was 104.4 degrees; and at noon, 106. During the previous night, she had been very delirious; and as the temperature rose, she became much worse. At noon (twentieth day), she was rapidly becoming comatose, could be roused, but with much difficulty, and had lost her sight. It was then determined to try the cold bath treatment. She was placed in the bath, and five minutes afterwards the temperature (in the rectum) was 106.2 degrees.

In half an hour, thirty-five minutes after entering the bath, the temperature had fallen to 102.2 degrees. Ten minutes later, she was romoved from the bath, and the thermometer showed 101.2 degrees, and thirty-five minutes afterwards, 99 degrees. For seven hours afterwards the temperature ranged between 100 and 102 degrees (in the axilla). Unfortunately there are no notes of the temperature of the bath, but Dr. R. thinks they commenced with a temperature of 90 degrees, quickly reduced by the addition of ice to 60 degrees. But a small amount of alcoholics was given while the patient was in the bath. As soon as the temperature began to fall, consciousness returned. There was no delirium, aud all the serious symptoms were removed during the day. The morning after the bath, the temperature was 100.6 degrees, and on the following morning it fell to 93.6 degrees, and never rose higher. From that time the patient rapidly improved and recovered.—*The British Medical Journal*, October 2, 1875.

Propylamin in Acute Rheumatism (*The Lancet*, November 6th, 1875).—Dr. Lee has treated twenty-eight cases of acute rheumatism with a solution of one gramme of propylamin and ten of sugar in one hundred and twenty grammes of peppermint water, of which a tablespoonful was given every two hours; altogether from three to five grammes was so taken by each patient, whose limbs were bandaged with cotton-wool and cardboard. All of the twenty-eight cases suffered from multiple joint affections; in fourteen cases the disease disappeared for the first time, in the other fourteen it was recurrent once or repeatedly. Five cases were complicated with slight, five with severe affections of the heart, one with scute œdema of lungs, and one with diptheria. All were restored to perfect health and military duty except one. The average duration of the illness in these cases wa^e 17.7 days per head; none was discharged until full recovery was proved by increased weight of body and gymnastic exercises. The effect of propylamin is summed up as follows: 1. The disease becomes very soon subacute, and remains so to the last. 2. The sedative effect on the nervous system is shown by decreased tension in the circulatory apparatus; pulse and respiration become slower, and high fever decreases within thirty-six hours. 3. With that first profuse, then more gentle perspiration, pain decreases very markedly. 4. The color of the skin acquires a peculiar grayish tint. (?) 5. Sleep quickly returns, and is not interrupted by pain. 6. With a cleaner tongue appetite returns fast. 7. The quantity of urine is not much increased; it is mostly clear and transparent, only slightly acid, and with little sediment. 8. All patients took the drug without dislike; it was never applied externally.—*Medical Times.*

"Case 2.—Mrs. L. C., Scotch lady; very stout and fleshy, fell in the water by the upsetting of a skiff, at 6 P. M. She was taken home in a carriage, and at 8 P. M., I was called in, when found her fearfully convulsed on the *left side only.*

I administered opiates, with dry friction, etc., when profuse perspiration set in. The next morning the left side was totally paralyzed, with the exception of a slight motion of the toes; face not affected—the tongue, however, could not be protruded straight.

Although from my experience of the former case with the bromide, I hesitated a similar treatment, because the lesion here must be below the pons varolii. However, after the effects of the opiates had passed away, she became quite restless. I commenced on 20 grain doses every hour; so that some days she took from 600 to 800 grains. On the tenth day she was dismissed as cured, having a return of the use of her limbs, with but a slight limping. Her arm, as she expressed it, "felt sleepy." Her mind shows no disturbance." The Doctor closes his paper with the following remarks: "Now as to the anatomical changes, it would be mere speculation. Yet, unless it should be with concussion that extravassation took place, a speedy action of with absorbents would at any rate prevent any great change taking place in the brain substance. Now, then, may it not be proper to infer that the action of the bromide is more on the lymphatic vessels, stimulating them, rather than on the nervous system? And, in any case, if the nerves be simply irritated, preventing sleep, will not sleep be restored when that irritant is removed? And the bromides will do it, if it stimulates the absorbents." (*The Cincinnati Lancet and Observer*, Oct. 1875.)

UTERINE ASTHMA.—(*The Practitioner*, December, 1875).—Mr. G. Waring-Curran calls attention to the variety of asthma not unfrequently met with, in those patients at a particular time of life who suffer from uterine tumors, and which seems slow in yielding to the ordinary remedies. Women with fibroid tumors of the uterus or its appendages appear to suffer more severely than those who have any other form of tumor, and the paroxysms of asthma are most severe during the menstrual period, which, in general, is excessive and prolonged. The result of his experience is that in such cases applications to the chest, inhalations, and smoking afford no relief, but the whole attention must be directed to the uterus. If the asthma precede the catamenia, the flow must be encouraged by a warm bath and a small dose of ergot. Should the discharge be excessive, it must be controlled, but not stopped, by judicious and well timed doses of the same drug; but that which will relieve the asthma at any stage is belladonna, applied locally and given freely internally. The extract is the most satisfactory and reliable preparation for external use, while the tincture may be given internally, combined with full doses of bromide of potassium. In the intervals iron and strichnia should be prescribed, and the iodine applied locally; in any instance where there is prolapse of the womb with a tumor behind, belladonna suppositories are useful. The extract of belladonna should not be cofined to the region where the tumor is situated, but the spinal nerves should get the benefit of a little spread on a piece of lint and applied along the lower dorsal and lumbar spine.

Alcohol in Glandular Swellingss—A new use for alcohol has been discovered by a German physician of Weinheim, Dr. Schwalbe, who, if we may credit the accounts which reach us, has reported one hundred cases of various forms of tumors and glandular swellings which he has successfully treated by the subcutaneous injection of the tincture of iodine. Subsequently, having injected the simple alcohol with equally favorable results, he concluded that the latter is really the effective agent. The subcutaneous injection of this fluid produces chronic inflammation of the connective tissue, by which it is caused to contract, and the pressure thus produced obliterates the lymphatics. In some cases he has used ether in connection with alcohol to dissolve fats, and in this way he reports that he has cured several cases of fatty tumors. But the most important discovery of all is that alcohol thus administered prevents the extension of the cancer to neighboring tissues and lymphatic glands. By injecting the alcohol the tumor may be isolated, as it were. The contractive connective tissue, then approaches the growth itself, presses upon it, stops the supply of blood, and causes it to disappear by atrophy. In connection with Dr. Hassee, Dr. Schwalbe claims that he has cured three cases of cancer of the breast in this way. We suppose that temporary cures are meant, as cancer is a disease of the blood, and is, therefore, constitutional, although locally manifested; and it is well known that the successful removal of cancer of the breast is very apt to be followed by the re-appearance of the disease elsewhere.—*American Artisan*.

SCROFULOUS PERSONS—METHOD BY WHICH THEY BECOME TUBERCULOUS.—Rindfleisch (*Ziemssen's Cyclopædia, Vol. V.*) illustrates his latest views as follows: If a scrofulous child is accidentally struck on the elbow, the joint may become inflamed. Such an arthritis, accompanied with pain and congestion, may last several months, then take on a fungous character, and the cavity of the joint becomes filled with pus. The cells of this purulent secretion degenerate, and their detritus remains in contact with the diseased synovial membrane, is absorbed, and there results from its absorbtion local and general miliary tuberculosis. If, in some other child, from bad food, a catarrhal inflamation of the small intestine is excited, the adenoid tissue of the mucous membrane becomes inflilfrated wich cells. These cells break down and are absorbed by the lymphatic vessels. Then there are produced miliary tubercles along the course of the lymphatics up to the mesenteric glands, and even beyond them, so that there is general tuberculosis. In the same way a scrofulous ophthalmia, an impetigo, or a scrofulous ozæna may lead to tuberculosis of the cervical lymphatic glands, and then of the entire body. A catarrh of the larynx, due to cold, may give rise to a scrofulous infiltration of the vocal cords, or the folds of the glotis. The cells of this infiltration may break down, and their debris excite, on account of the unfavorable condition for absorption, an eruption of miliary tubercles in the vocal cords, followed by tubercular infliltration.—*Detrot Review of Medicine*.

MILK DIET—IS IT INJURIOUS IN DIABETES?—Dr. Donkin (*British Medical Journal*, October 2, 1875) contends that milk sugar differs so essentially from cane sugar, grape sugar, and pure diabetic sugar, that it is not converted into it, and does not appear again in that form in the urine. Belonging to a different class of sugars, milk does not undergo alcoholic fermentation in contact with yeast, and in addition, it does not precipitate the oxide of copper when treated with the reduction test. On the other hand, it is subject to lactic fermentation by the action of ferments. On account of these intrinsic differences, lactose, as an ingredient of milk, cannot undergo the same metamorphic changes as glucose in the processes of digestion and assimilation in health, (nor be converted into it in diabetes,) its conversion into lactic acid being direct and immediate, not by intermediate changes through which the latter pass into this substance. For this reason, which is perfectly intelligible, milk sugar, unlike vegetable glucos, is assimilated, and the reverse of injurious in diabetes. To give skim-milk a fair trial, no other food must be allowed to mix with it in the stomach. If it cannot be made the exclusive diet, care must be taken that the milk be entirely absorbed ere other food be swallowed.—*Detroit Review of Medicine.*

Boracic Acid in the Treatment of Ringworm.—Surgeon-Major Watson reports, in the *Indian Medical Gazette,* that he he has lately employed boracic acid with very great success as an external application in the treatment of dermatophyta or vegetable parasitic diseases of the skin. He was induced to try this remedy from witnessing its employment as an antiseptic in the Edinburgh Infirmary wards. The diseases in which he has hitherto used boracic acid have been the different forms of tinea (T. tonsurans and circinata), and in that very troublesome form of the disease which affects the scrotum and inner side of the upper part of the thighs of many Europeans in India. Dr. Watson declares that the external application of a solution of boracic acid acts like a charm in such cases. An aqueous solution of boracic acid of a drachm to the ounce, or as much as the water will take up at ordinary temperatures, is employed. The affected parts should be well bathed with the solution twice daily, some little friction being used, and should not be wiped off, but allowed to dry on the part. The remedy is said to be so simple, cheap, and efficacious, that it has only to be once used to be preferred to all other remedies of the same class.

The Treatment of Vomiting, or Pregnancy.—The interesting paper in the *Journal* of November 6th, by Dr. Copeman, on obstinate vomiting connected with pregnancy, has induced me to bring into more prominent notice a remedy which, during the last four years, I have found of great service in relieving, if not in subduing, the ordinary nausea and sickness of early pregnancy. This remedy is dilute phosphoric acid in doses of from thirty to sixty minims in a wine-glass of water two, three, or four times a day, as may be required. It is of special value in asecs where the nausea becomes extreme at the sight of food, as a dose may be easily taken before meals. Amongst all the remedies for this particular discomfort, I have found none so uniformly efficacious. It may act by powerfully stimulating the nerves of the stomach, or as a corrective (if, as is asserted, the vomitings of pregnancy are alkaline), or in both ways. It is very pleasant to the taste, and I have always found that patients who have taken it in one pregnancy invariably send for the acid when they find themselves in an interesting condition again.—*Dr. Fairbank, British Medical Journal.*

Ganglion of the Wrist.—The young lady now presented has a bursal or thecal tumor, sometime called a ganglion, on the back of the left wrist. This has been caused by a deposit of inflammatory new material; a blow, or some like cause has excited some slight inflammation, in consequence of which the sheath, becoming inherent to its tendon, has formed a pouch, or bag, in which the natural secretion accumulated. The fluid contained is thick and jelly-like, similar to the gum that exudes from a cherry-tree. Possibly, now and then, the affection may exist as a congenital defect, but it generally occurs as I have described.

The treatment is simple. The older surgeons used to rupture this cyst, and disperse it into the cellular tissue by a blow with a weight or a book; the Bible was the favorite for the purpose, on account of a supposed special virtue in this direction. I will introduce a tenotome under the skin, some distance from the tumor, scarify it and liberate its contents; the sac will subsequently be obliterated by inflammation, encouraged by painting the skin over it with iodine, and applying pressure; an old-fashioned cent, or silver quarter, makes a good compress. The dressing will remain on for three or four days, and then be temporarily removed to make a fresh application of tincture of iodine. If I were not a modest man I might lay claim to originality for this method of treatment, as I have never seen it described anywhere: it is not in any of the books, and I have used it with great success for many years.—*Prof. Gross Medical and Surgical Reporter.*

Treatment of Tape-Worm by Creosote.—Mr. Brickwell states that about fifteen years ago a man discharged from the army with lung disease and tape-worm came under his notice, when it occurred to him that the destructive properties of creosote to the lower grades of animal life might be made available for killing or so weakening the vitality of intestinal worms that they could easily be got rid of. He therefore gave him some three times a day, shortly after meals, for six days, and on the seventh a dose of castor oil and turpentine, which brought away a worm twelve yards long. He has since tried creosote for destroying round worms with great success. In one case a large mass of more than a hundred worms of all sizes came away, and the patient has not been further troubled by them. He has not succeeded so well with the troublesome thread worms of adults, but has some doubts if the remedy were fairly tried.—*Medical Times and Gazette*, and *Practitioner.*

A Surgical Nail.—The idea of employing an artificial nail for surgical purposes is by no means novel. Chassignac invented one, which he however soon abandoned, as its form necessarily limited the sensibility and freedom of motion of the finger. And yet the importance of such a nail cannot be denied, especially where the surgeon is working in the deeper tissues in the neighborhood of the vessels, where he is unwilling to use the knife and where the strength of the natural nail is insufficient to tear the tissues.

The nail exhibited by Motais to the Paris Academy of Medicine (*Gaz. des Hop..*) seems to be exactly adapted to the purpose; it allows free motion of every joint of the finger and does not cover the sensitive extremities. It consists of a narrow ring at the base of the third phalanx to which a small steel plate is attached, which corresponds with the natural nail and lies upon it. Its free border is curved and extends about one millimetre beyond the pulp of the finger. Its inner surface is, of course, hollowed for the reception of the natural nail. By means of this instrument Motais has removed three uterine polypi.—*Schmidt's Jahrbucher.*—*The Clinic.*

Antidote for Poisoning by Rhus Tuxicodendrum.—Dr. Williams says: In the fall of 1873, while gathering herbs for pharmaceutical purposes, I accidentally brought my left forearm into contact with the end of a freshly cut shoot of poison oak. Not being, as I thought, susceptible to its toxical effects, I paid but little attention to the occurrence. A few hours afterwards, during which time an intense itching sensation was experienced, I noticed the usual tumefaction and rapidly appearing erysipelatous rash. Having had considerable experience in the use of hoposulphite of soda in various forms of disease, I concluded here was a test for its antidotal power, if it possessed any, and I at once applied a saturated solution to the affected part. The effect was marvelous, positively magical, as in three or four hours' time every symptom of the disorder had disappeared. Since that time I have used it with like result, not only in cases of rhus poisoning, but also in erysipelatous inflammation. Both internally and locally it proves itself a reliable remedy.—*Druggists' Circular.*

Alcoholic Phthisis.—Dr. B. Richardson (*Medical Times and Gazette,* August 7, 1875) lays emphasis upon a form of consumption, due to abuse of alcohol. Its subjects are in middle life—males of robust built, and naturally healthy. They sleep, eat and drink pretty well; are active in body or mind, and enjoy life. They drink of any alcoholic drink they may meet, and with remarkable tolerance. As a rule, they can only work on a daily excess of alcohol, which excess must be increased if extra work is to be done. The symptoms of phthisis do not always begin when the patient is indulging in alcohol—he may have for some time become abstemious. The disease begins with pain in side, sharp in the onset, but later on continuous, and as it subsides, there is difficulty in breathing. Then follows hæmoptysis, and the fatal end is near. Medicine affects the disease but little, the best diet fails, change of air accomplishes naught.—*Detroit Review of Medicine.*

INUNCTION.—Dr. W. R. Fisher (*Medical Record*, March 27, 1875,) in an interesting discussion of inunction, reaches the following conclusions:

(1) The oils and fats are essential to growth and development.

(2) Inunction is a valuable method of introducing these nutritive materials into the body, to be used when the stomach will not tolerate the oils, or when we wish to produce special effects by this method of application.

(3) The vegetable oils are more suitable than the animal oils and fats for the purposes of inunction.

(4) The oils and fats are chiefly useful in those diseases and derangements of the system in which wasting enters as an important factor. Hence, we may expect that their employment by inunction will be specially beneficial in scrofula, constitutional syphilis and rickets, in convalescence from acute diseases, in nervous affections, and in many chronic diseases.

(5) Children are more suitable subjects for inunction than adults, partly because their susceptibility to external influences is much more acute than that which is exhibited at later periods of life, and partly because the applications are more easily made than in adults.—*Detroit Review of Medicine.*

A NOVEL EXPEDIENT IN A DIFFICULT LABOR.—To illustrate what a quick witted man may do in an emergency, Dr. Cotting gave the account of a sea-captain who was crossing the Atlantic with a load of immigrants, one of whom was a woman in labor. At the end of the first day he consulted his medical library without getting any satisfactory information. At the end of the second day the woman was getting exhausted, the pains were diminishing, the scalp protruding, "everything blocked up," general condition of things deplorable. Appealed to in this emergency, the captain placed the woman on her side, flexed the opposed thigh forcibly upon her abdomen, placed his knee against the lower end of the sacrum, at the same time telling her to strain away for once and all, which being done, the child was immediately born.—*Boston Med. and Sur. Jour.*, Jan. 28, 1875, p. 103.

HEADACHE, NERVOUS.—Five grains of croton-chloral every three hours, or even oftener, will give, in most cases, considerable relief. I need hardly say, that the drug does not entirely free the patient from her attacks; but, in one or two days, the pain ceases to be continuous, then the attacks recur, but only once or twice a week, the interval gradually extending till an onset occurs only every week, then about every fortnight, or even longer, till the illness assumes its old type and periodicity. In some cases a week's treatment suffices to bring back the headache to its original type of an attack once in three or four weeks. Then the croton-chloral appears to be far less serviceable, manifesting but slight effect on the periodical attacks. In many cases of ordinary periodical headache, the patients say that, in the milder form, the drug distinctly lessens the severity and duration, but in the severer forms, it is without effect, even when sickness is absent. In those cases accompanied by severe vomiting and recthing croton-chloral is useless, being speedily rejected.

Croton-chloral, I have fonnd, will relieve the slight attacks experienced by some delicate and nervous women after any slight fatigue or excitement.

ACTION OF ALKALIES UPON THE COMPOSITION OF THE BLOOD.—Pupier (*Comptes Rendus—Edinburg Medical Journal*, October, 1875) gives the results of an experimental investigation of this subject:

1. The administration of alkalies in a state of health tends to augment the number of red corpuscles, to increase the temperture and weight of the subject, and to favor trophic phenomena.

2. In a diseased condition of the tissues of a considerable extent, they produce anæmia by aiding in morbid changes.

3. On the one hand they increase physiological function, and on the other, they stimulate pathological processes. A case is cited in which a man, for twenty-nine years, took a daily dose, of three hundred grains of anhydrous bi-carbonate of soda, without anæmia or loss of health.—*Detroit Review of Medicine.*

Chloroform in the Surgery of Infants.—Dr. Bergeron, of Paris, (*London Obstetrical Journal*, June, 1875,) from his personal experience upon this subject, concludes that—

1. Chloroform in the infant is endowed with an almost absolute harmlessness.

2. The child, not having come to the age of reason, nor feeling any moral emotion, suffers from no apprehension of the dangers to which it may be exposed, nor experiences the apnœa which produces so much terror, and is, the author imagines, a most important cause of death supervening suddenly during the administration of chloroform.

3. Chloroform may be administered to the infant from the first day of its birth.

Treatment of Carbuncle by Strapping.—Dr. Zeigler (*Philadelphia Medical Times*) reports two cases successfully treated, after the manner originally proposed by Dr. J. Ashurst, Jr. The indication is to make concentric pressure by adhesive strips, three-fourths of an inch in width, tightly drawn over the suppurating tumor in different directions, leaving a small outlet in the centre for the discharge. The result is described as highlv satisfactory, the diseased structures rapidly diminishing under the pressure. The plan appears to be adapted to the suppurating stage of carbuncle.—*Pacific Medical & Surgical Jour.*

Lime Glycerine for Burns.—This remedy is as follows:

| | | |
|---|---|---|
| ℞ Oxidi, calc., | gram. | iij. |
| Glycerin., | " | cl. |
| Sp. æther chlor. | " | iij. |

This preparation has been used by Laub for several years with great success.

Charpie is to be dipped in the mixture and placed over the burned surface; it is then covered with a thin sheet of gutta-percha, and then a layer of charpie is added, the whole to be surrounded with a loose bandage. It ts very important that the charpie should be closely applied to the entire burned surface. The pain ceases almost instantly, and the sore heals very rapidly. *Hospitalstidende* and *Nordiskt Med. Arkiv.*

Diphtheria.—Dr. Hauser says, I wish only to tell, in the plainest style, and fewest possible words, how I have cured it, in every case I have treated. Here is the plan:

R. Nicotiana tabacum, opt................
Piper rubrum, aa.........................drachm ij.
Sodii Chlor..................................ounces ij.

Pour boiling water on these articles, about two gills or more. Mop the tonsils and neighboring parts frequently, severe as it will be, until relief is obtained. Be bold, and fear not; nay, "doubt not." If half a grain of solid carbolic acid be added to the above, all the better. Mop when the decoction is blood-warm.—*Charleston Medical Journal and Review.*

Strychnia as an Anti-Emetic.—Dr. Thomas E. Dupuis reports in *The Canada Lancet,* a case of obstinate vomiting in a delicate female, which was absolutely uncontrollable, and continued until the most extreme prostration had occurred. After nearly all other remedies had been tried unsuccessfully, the following solution was given in drachm doses every two hours: R. Liquoris strychniæ, M. xx; aquæ, ounces iv. The effect was very sudden and decided; the vomiting stopped entirely within a few hours, and the patient soon recovered.—*New Remedies,* January, 1875.

Sore Nipples.—I have had a large obstetric practice as an English physician, and have *never* had a bad case of sore nipples. For many years, when the nipples became slightly sore, I at once applid *zinc shields;* but of late years, instead of allowing the zinc to combine with the lactic acid of the milk, I have applied a preparation of the sulphate of zinc and lactic acid (in fact lactate of zinc) and glycerine with starch, between the times of suckling. I think if you try this, you will find it unfailing, and not only a "prophylactic," but a *specific* in the true sense of the term.—Fleischmann.—*Canada Medical Record.*

Solution of Camphor in Erysipelas.—In the *Gazette Medica da Bahia* it is said that a few drops of a solution of equal parts of gum camphor and ether, applied from time to time to an erysipelatous surface will, in the majority of cases, effect a cure. —*New Remedies,* Jan. 1875, p. 15.

